# Surface Testing of Dental Biomaterials—Determination of Contact Angle and Surface Free Energy

**DOI:** 10.3390/ma14112716

**Published:** 2021-05-21

**Authors:** Aneta Liber-Kneć, Sylwia Łagan

**Affiliations:** Department of Applied Mechanics and Biomechanics, Faculty of Mechanical Engineering, Tadeusz Kosciuszko Cracow University of Technology, 37 Jana Pawla II Av., 31-864 Cracow, Poland; sylwia.lagan@pk.edu.pl

**Keywords:** surface free energy (SFE), contact angle (CA), dental materials, enamel, adhesion

## Abstract

The key goal of this study was to characterize surface properties of chosen dental materials on the base on the contact angle measurements and surface free energy calculations. Tested materials were incubated in the simulated oral environment and drinks to estimate an influence of conditions similar to those in the oral cavity on wetting and energetic state of the surface. Types of materials were as follows: denture acrylic resins, composite and PET-G dental retainer to compare basic materials used in a prosthetics, restorative dentistry and orthodontics. The sessile drop method was used to measure the contact angle with the use of several liquids. Values of the surface free energies were estimated based on the Owens–Wendt, van Oss–Chaudhury–Good and Zisman’s methods. The research showed that surface wetting depends on the material composition and storage conditions. The most significance changes of CA were observed for acrylic resins (84.7° ± 3.8° to 65.5° ± 3.5°) and composites (58.8° ± 4.1° to 49.1° ± 5.7°) stored in orange juice, and for retainers (81.9° ± 1.8° to 99.6° ± 4.5°) incubated in the saline solution. An analysis of the critical surface energy showed that acrylic materials are in the zone of good adhesion (values above 40 mJ/m^2^), while BIS-GMA composites are in the zone of poor adhesion (values below 30 mJ/m^2^). Study of the surface energy of different dental materials may contribute to the development of the thermodynamic model of bacterial adhesion, based on the surface free energies, and accelerate the investigation of biomaterial interaction in the biological environment.

## 1. Introduction

The biomaterial surface plays a key role in interaction between the biomaterial and the biological environment. In the oral cavity, surface of the teeth and dental materials are subjected to the phenomena of adhesion and removal of plaque [1,2]. When bacteria adhere on the biomaterial surface, they proliferate and form a biofilm [3]. The factors which influence bacterial adhesion are critical and revealed by many studies. Bacterial adhesion cannot be described by one general mechanism [4]. The biochemical approach highlights that pellicle which is a thin layer of organic material (saliva glycoproteins, phosphoproteins, proteins, enzymes and receptors for adhesins bacterial) is essential for bacterial attachment to surfaces [4]. The physicochemical mechanisms of bacterial adhesion involve a thermodynamic model based on the interfacial free energies of liquids and interacting surfaces. The classical Derjaguin, Landau, Verwey, Overbeek (DLVO) theory explains bacterial adhesion as the effect of Lifshitz–Van der Waals, acid–base and electrostatic interactions [3,4]. Numerous factors regarding materials have been identified to influence an oral biofilm formation such as a surface roughness, surface free energy and surface chemical composition [3,4,5,6].

Characterizing the surface properties of a biomaterial, before putting it in a biological environment, provides information about its different features such as topography, roughness, surface energy, etc., in order to find a correlation with the cell behavior [7]. Different techniques and analytical tools are used to characterize biomaterial surface. Chemical and morphological features of a surface are obtained by microscopic methods such as scanning electron microscopy (SEM) and spectroscopic techniques, e.g., X-ray photoelectron spectroscopy (XPS) and secondary ion mass spectroscopy (SIMS) [8]. To characterize surface topography and roughness, atomic force microscopy (AFM) can be used [5]. Typically, surface roughness is measured using a profilometer, which yields a two-dimensional cross section of the surface from which roughness parameters are taken [3]. The surface topography, together with surface roughness and surface pattern, influences on a cell adhesion [9]. A variety of topographically patterned surfaces affect their cell attachment efficacy in different ways. The size, spacing, and shape of a surface features plays an important role in cell adhesion [10]. In vitro studies have demonstrated that increased surface roughness promotes bacterial adherence [3]. Theoretically, the roughness of the surfaces of dental materials should be reduced below 0.2 μm [4]. Knowledge about controlling cell adhesion helps to design surfaces of special biomaterials. Other surface feature is wettability of solid surface evaluated by a contact angle measurement. Contact angle (CA) measurement also allows to calculate surface free energy (SFE), providing information about the polar or nonpolar nature of the interactions at the interface liquid/solid and the hydrophilic or hydrophobic character of a surface [11,12]. Wettability determines rearrangement of the functional groups at the surface of biomaterial in contact with a cell [12,13]. Bacterial adhesion is more likely on hydrophilic surfaces showing high values of surface free energy than of hydrophobic surfaces [9]. For polymers, values of water contact angles between 40° and 70° are reported as most suitable for cell adhesion [14]. The study conducted by Quirynen et al. showed a correlation between value of free surface energy versus quantity of plaque. Areas of low value of SFEs were characterized by a less mature plaque supra- and subgingival [6]. However, it is difficult to clearly state the range of the contact angle for which cells effectively adhere onto material surface. The cell adhesion onto material surface is a complex process, and it is affected by a variety of factors including cell types, surface wettability, roughness, topography and chemistry. The external conditions such as a methods, time and environment of conducted tests can also cause results of different studies not to be consistent [14,15,16,17].

The aim of this article is to compare surface properties of chosen dental biomaterials and medical devices incubated in the simulated oral environment and drinks based on a contact angle measurement and surface free energy calculations. Besides comparison of materials’ surface hydrophilicity and hydrophobicity, special content will be made to estimation of degree of interaction between a material exposed to a simulated biological environment and a living organism with the use of surface free energy and critical surface tension. The amount and composition of the bacterial plaque on dental materials is different and dependent on the type of material [12,18]. Conducted analysis can show if tested groups of materials (all polymer based) differ in values of SFE and critical surface tension as factors of adhesion. Comprehensive study of several dental materials can show these with higher SFE, which influence on tendency to adhere dental plaque during their initial immersion in aqueous media [9]. For applications in which a biomaterial surface will be in contact with a liquid phase, such as dentures and orthodontic retainers, it is not only important to know the surface characteristics under normal experimental conditions but also to determine the effects of exposure to the liquid medium. Therefore, tested materials were exposed to several medium of interest to determine a surface characteristic under conditions that mimic as closely as possible the conditions of use. Dental materials behavior in oral environment is a base for proper indication for their use.

## 2. Materials and Methods

### 2.1. Materials

Several dental materials such as acrylic resins, resin composites and orthodontic retainers were used in this study (Table 1). The materials selected for research are popular and widely used in the areas of restorative dentistry, prosthetics and orthodontics. Chosen materials were polymer based. In the group of prosthetics materials, acrylic resins differing in conditions of polymerization (cold or heat curing) and thermoplastic polyolefin were tested. As restorative material, a universal light-curing BIS-GMA resin was used. Acrylic resin and composite samples were prepared by using volume ratio of components and polymerization conditions according to the manufacturers’ instructions. Three types of samples were prepared: a beam (30.1 ± 1.1 mm long, 10.5 ± 0.8 mm wide, 4.7 ± 0.2 mm thick, the average weight was 1.7 ± 0.1 g), a medical device (retainers) with average weight 1.6 ± 0.6 g, and enamel of swine tooth. Dental enamel from swine molars was employed as the control. Swine molars were obtained as waste from a local slaughterhouse (F.H.P. Zet-Pol, Myślachowice, Poland). Retainers were of removable type, made of polyethylene terephthalate glycol, dedicated for patients for time of treatment from 6 to 12 months. Samples were tested as delivered (dry state) and after exposure to the simulated oral environment including different fluids and substances for the hygiene of dentures. The type of environment used, temperature and the time of exposure were shown in Table 2. A total of 62 materials’ samples and four samples of dental retainers were tested.

### 2.2. Methods

The measurements were performed with a sessile drop method [19,20,21,22] using the optical goniometer (Advex Instrument, Brno-Komín, Czech Republic) and the corresponding software SeeSystem. The following standard liquids with a well-known value of surface tension were used in the tests for contact angle measurements: diiodomethane and alpha-bromonaphtalene (Merck, Warsaw, Poland), glycerol and ethylene glycol (Chempur, Piekary Śląskie, Poland), distilled water (Biomus, Lublin, Poland). A liquid drop of 0.5 µL was dropped perpendicular to the material surface, with the use of micropipette Vitrum (VITRUM/VWR, Stribrna Skalice, Czech Republic), and the drop profile image was captured. The software automatically calculated the value of contact angle from the drop profile image based on the height and width analysis of the drop. Each biomaterial sample was subjected to 10 measurements. The final contact angle, used for analysis, was the average of ten values. The surface free energies of the different materials were calculated using the Owens–Wendt (OW) [12] and van Oss–Chaudhury–Good (vOCG) [20,21,22]. The Zisman’s approach was used to obtain values of the critical surface tension [23]. The theoretical basis of the methods used were described in the next section.

#### 2.2.1. Contact Angle

**Figure 1:**
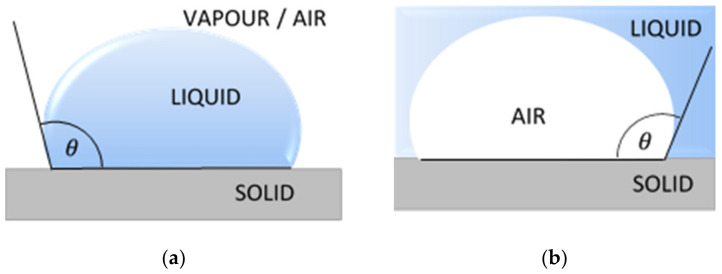

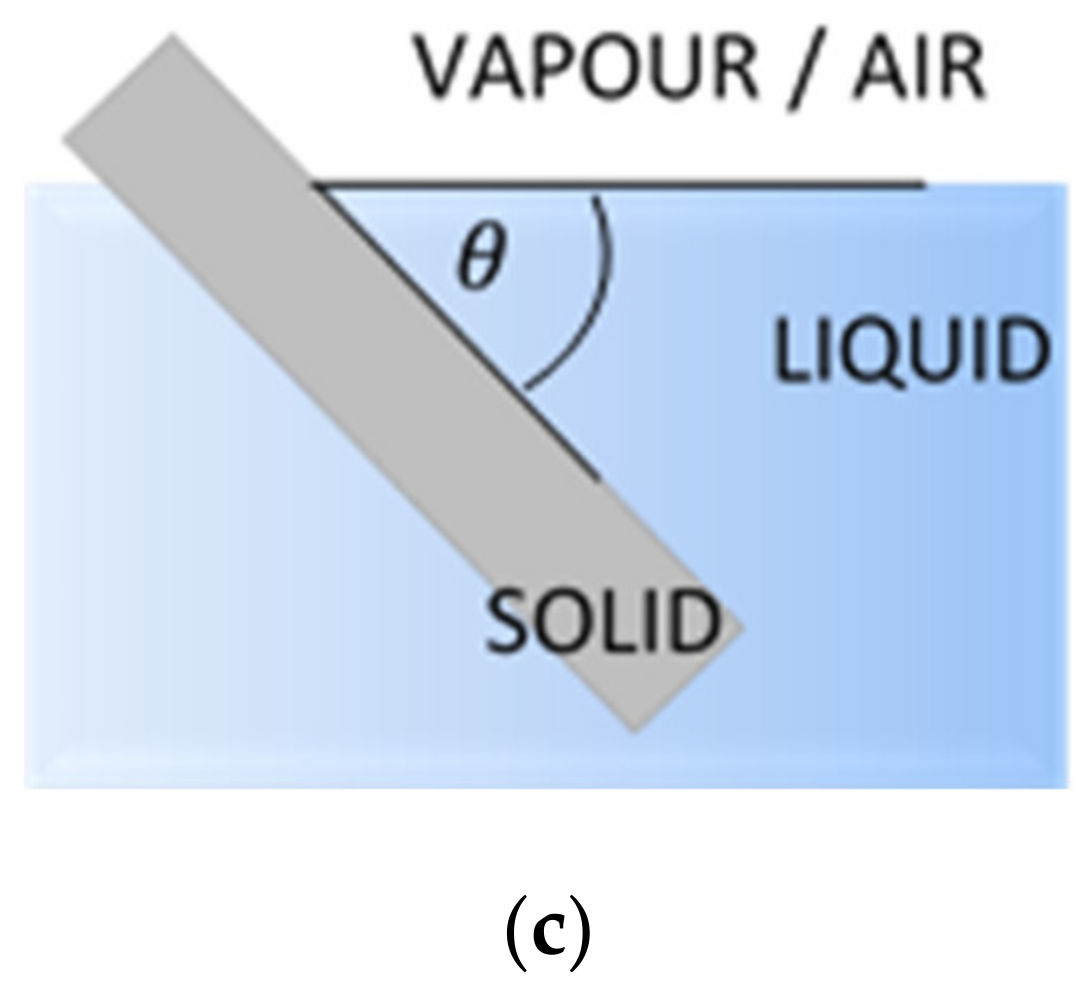
Common methods of the contact angle measurements: (**a**) sessile drop; (**b**) captive bubble; (**c**) tilting plate. θ is a contact angle.

The measurement techniques of contact angle can be classified into two main groups, the direct optical methods and the indirect force methods. The most common direct methods are the sessile drop, the captive bubble and the tilting plate (Figure 1). Indirect methods include tensiometer and geometric analysis of the shape of a meniscus. Calculations based on measured contact angle values yield are an important parameter of the solid surface tension, which quantifies the wetting characteristics of a solid material surface [24].

**Figure 2:**
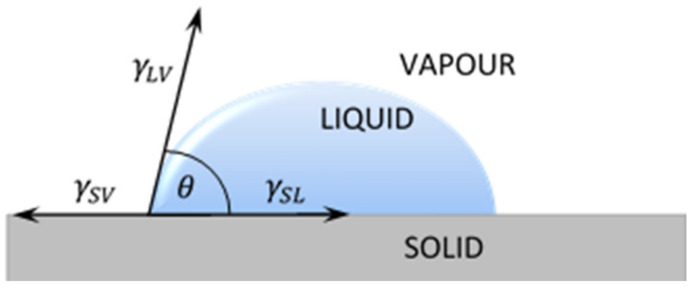
A liquid droplet remaining in equilibrium state with a plane solid surface: $\gamma_{\mathrm{SL}}$ - free surface energy between solid and liquid; $\gamma_{\mathrm{SV}}$ - free surface energy between solid and vapor; $\gamma_{\mathrm{LV}}$ - free surface energy between liquid and vapor.

The most popular technique of contact angle measurement is a direct measurement of the tangent angle at the three-phase contact point on a sessile drop profile (Figure 1a). This optical method needs small amounts of liquid and small surface of solid. The contact angle of a liquid drop on an ideal horizontal solid surface is determined by the mechanical equilibrium of the drop under the action of three interfacial tensions of liquid (Figure 2).

The Formula (1) is usually referred as Young’s equation, where γ_LV_, γ_SV_, γ_SL_ represent the liquid–vapor, solid–vapor and solid–liquid interfacial tensions, respectively, and θ is measured contact angle of liquid.

(1) $$\gamma_{\mathrm{LV}}\cos\theta =\gamma_{\mathrm{SV}}-\gamma_{\mathrm{SL}}$$

The Young’s equation applied to a concrete liquid–solid system, describes three thermodynamic parameters and the contact angle. However, there exist many states of a drops on a surface solid, and the observed contact angles are usually not equal to θ. The liquid migrates on surface and wet the new area of surface of the solid, so the phenomenon of wetting is not a static state.

#### 2.2.2. Surface Free Energy Calculation

The surface free energy is a kind of an attraction force of the surface which cannot be measure directly. Surface free energy determines how the solid behaves in contact with other materials. Contact angle measurement in different measurement liquids with different surface free energies are used in calculations according to several approaches [25]. In this study, three theories such as Owens–Wendt’s, van Oss–Chaudhury–Good’s or Zisman’s were used for surface free energy calculations.

##### Owens–Wendt Method

The Owens–Wendt model considers the geometric mean of the dispersive and polar parts of the liquid’s surface tension and the solid’s surface energy [26]. This method assumes that surface free energy (γ_S_) is a sum of two components: polar (γ_S_^p^) and dispersive (γ_S_^d^) (2):

(2) $$\gamma_{S}=\gamma_{S}^{d}+\gamma_{S}^{p}$$

To determine the polar and the dispersive components of the SFE, the measurements of the contact angle onto samples surface must be conducted with two measuring liquids. The SFE of the measuring liquids used in the test is known, including its polar and dispersive components. Typically, the tests are carried out with distilled water as the polar liquid and diiodomethane as the nonpolar liquid. Polar and dispersive component of solid’s SFE is calculated from the Formula (3) by creating a system of equations (one with data for a polar liquid and the second with data for a nonpolar liquid).

(3) $$\frac{1}{2}\left(1+\cos\theta \right)\gamma_{L}=\sqrt{\left(\gamma_{S}^{d}\gamma_{L}^{d}\right)}+\sqrt{\left(\gamma_{S}^{p}\gamma_{L}^{p}\right)}$$

where $\gamma_{S}$-surface free energy of tested material, $\gamma_{S}^{d}$-dispersive component of SFE of tested material, $\gamma_{S}^{p}$-polar component of SFE of tested material, $\gamma_{L}$-surface free energy of measurement liquid, $\gamma_{L}^{d}$-dispersive component of SFE of a liquid, $\gamma_{L}^{p}$-polar component of SFE of a liquid and θ-measured contact angle.

The Owens–Wendt is one of the most common methods for surface free energy calculations.

##### Van Oss–Chaudhury–Good Method

According to the approach of the van Oss–Chaudhury–Good [27], the surface free energy of a solid (γ_S_) is the sum of apolar Lifshitz–van der Waals (γ_S_^LW^) and polar acid-base interactions (γ_S_^AB^) (4):

(4) $$\gamma_{S}=\gamma_{S}^{LW}+\gamma_{S}^{AB}=\gamma_{S}^{LW}+2\sqrt{\gamma_{S}^{+}\gamma_{S}^{\_}}$$

where and γ_S_^+^, γ_S_^-^ represent the polar components (acid–base).

Different components of the solid, the liquid surface free energies, and the contact angle are related by this Equation (5):

(5) $$\frac{1}{2}\left(1+\cos\theta \right)\gamma_{L}=\sqrt{\gamma_{S}^{\mathrm{LW}}\gamma_{L}^{\mathrm{LW}}}+\sqrt{\gamma_{S}^{+}\gamma_{L}^{-}}+\sqrt{\gamma_{S}^{-}\gamma_{L}^{+}}$$

In order to solve this equation, three unknown parameters γ_S_^LW^, γ+, γ− must be found. Thus, the contact angle measurement must be done with three different measurement liquids (one non-polar and two polar). This theory, sometimes also called acid–base theory, is the second most used surface free energy theory. It has especially been utilized to look at interactions of proteins (and other biopolymers) with hydrophobic solids [25].

##### The Zisman’s Method

**Figure 3:**
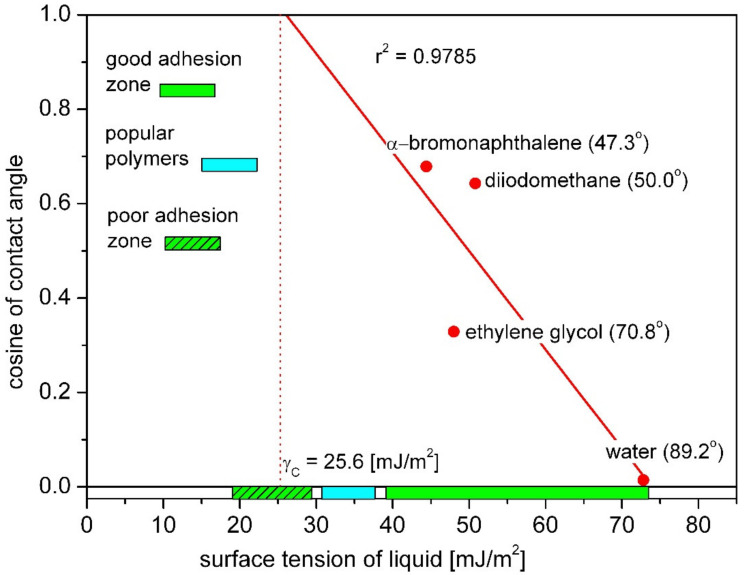
Zisman’s plot for PTFE with obtained value of critical surface free energy. Zones of surface energy were marked green according to [28,29].

The Zisman’s method is used to determine the so-called critical surface energy (γ_C_), which is the surface tension of the liquid needed to completely wet the solid (in that case the contact angle of solid is zero). This critical surface tension value differs from the surface free energy of the solid and is not divided into dispersive and polar components. In a contact angle measurement, several liquids from a given homologous series are used. On the basis of contact angle values, a plot is generated having the surface tension of the liquid in x-axis and cosθ in *y*-axis. Straight line is fitted to these measurement points and extrapolated to point cosθ = 1 which will give the critical surface tension value for the surface [23]. An exemplary Zisman’s plot made on the base on the contact angle values measured with the use of several liquids for polytetrafluoroethylene (PTFE) was shown in Figure 3.

The Equation (6) of the straight line can be determined in a defined coordinate system in which b is the directional coefficient of the line.

(6) $$\cos\theta =1+b\left(\gamma_{C}+\gamma_{L}\right)$$

Using the Equations (1) and (6), for tested material, the relationship between surface free energy $\gamma_{S}$ and critical surface free energy $\gamma_{C}$ can be given as (7):

(7) $$\gamma_{S}=\frac{{\left(b\times \gamma_{C}+1\right)}^{2}}{4b}$$

In the Zisman’s plot (Figure 3), the surface tension ranges corresponding to good and poor adhesion were shown. The zones were determined on the basis of research on interactions between polymer vascular implants and tissues reported by Baier [28]. In the late 1960s, Robert Baier investigated the role of surface energy of biomaterials in thrombogenesis and proposed the correlation of the degree of biological interactions with the critical value of surface free energy. The surface which indicated least retentive of depositing proteins was identified by the bioengineering criterion of having measured critical surface tension between 20 and 30 mN/m [28,29]. These studies showed that materials characterized by high values of the critical surface tension (above 40 mJ/m^2^) exhibit the best tissue adhesion. Poor tissue adhesion was observed for materials with low critical surface tension values (below 30 mJ/m^2^). Critical surface tension also influences the clotting time of blood surrounding the biomaterial [28,29].

## 3. Results

**Figure 4:**
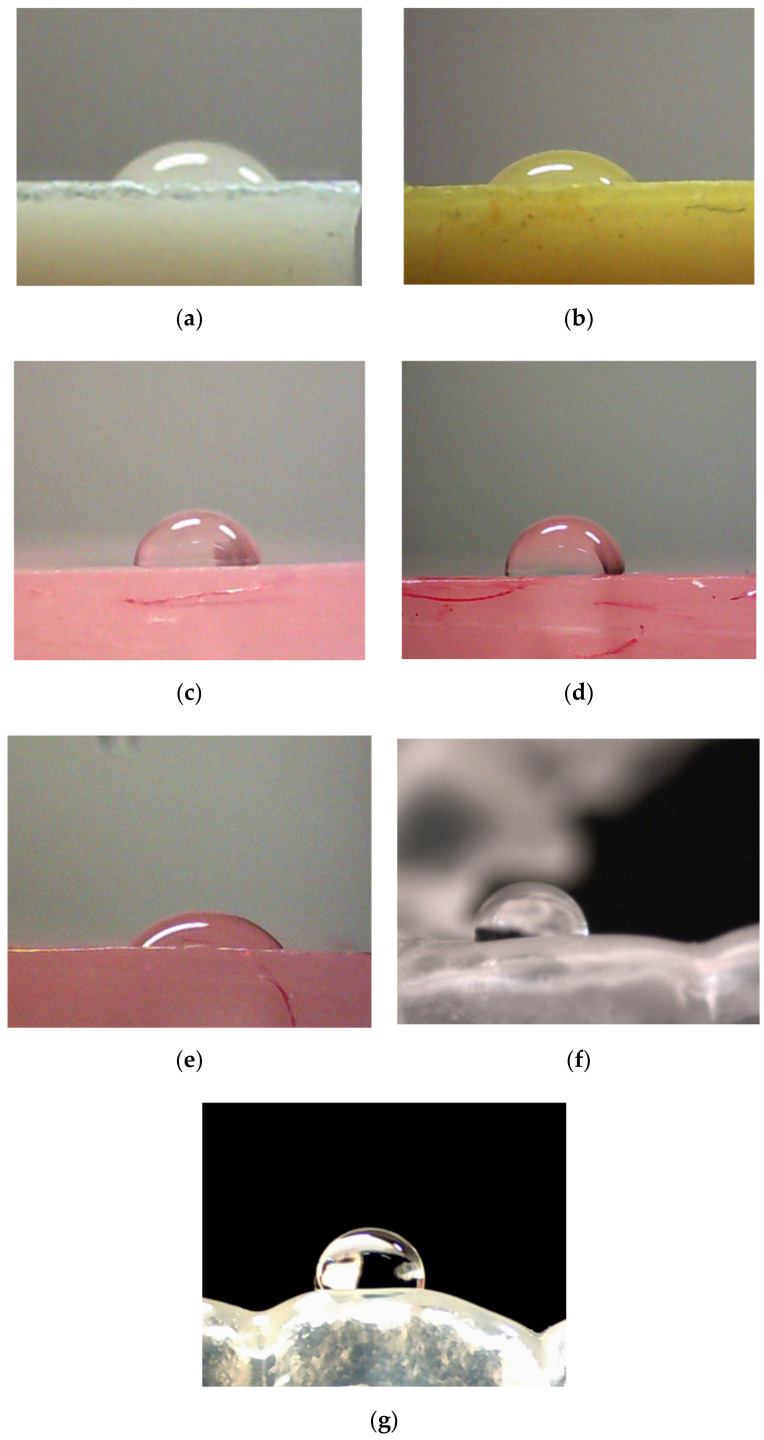
Exemplary images of a water drop onto surfaces of tested materials in the initial state and after incubation: (**a**) Vip Esthetic; (**b**) Vip Esthetic stored in orange juice; (**c**) Villacryl H Plus; (**d**) Villacryl H Plus sored in Corega (14 days); (**e**) Villacryl H Plus stored in Prokudent (14 days); (**f**) dental retainer; (**g**) dental retainer stored in water.

The values of contact water angle for tested materials in the initial state and after incubation in the simulated biological environment were presented in Table 3. Hydrophilic character of the tested materials’ surface can be seen but the surface of retainers and Vilacryl SP is slightly hydrophilic. It can be observed, that contact angle depends on the material composition and type of medium used for incubation. After incubation in saline solution, slight decrease in the value of contact angle for denture materials (Vertex and Flexite) was observed. The incubation liquid, which significantly influenced on decreasing the value of contact angle for acrylic resin (Villacryl SP) and BIS-GMA resin (Vip Estetic) was orange juice. After 14 days of exposure, contact angle value decreased by about 20% compared to value for materials in the initial state. The increase of the contact angle value was observed for acrylic resin treated with solution of fixing cream and retainer incubated in saline solution and water. For retainer character of the surface changed to hydrophobic. In Figure 4, exemplary images of water drop onto surface of tested materials in the initial state and after incubation were presented.

Measured values of contact angle for materials in the initial state and after incubation were used in calculation of the surface free energy according to the Owens–Wendt and van Oss–Chaudhury–Good models. The results of calculations were compared in Table 4. The comparison of the SFE values of the tested materials showed that acrylic resins have similar values in the range from 43.1 to 48.1 mJ/m^2^ (OW) and from 42.8 to 47.0 mJ/m^2^ (vOCG). Moreover, for acrylic resins, the total SFE values calculated according to the two models are within a similar range. Among the tested materials, the composite material is characterized by the energetic state of the surfaces closest to the pig enamel (OW model). The energetic state of surface of the tested composites happened to be stable after incubation in an orange juice, despite decrease of contact angle value. For denture acrylic resins, increase of the SFE value was observed after incubation in an orange juice. Incubation conditions such as saline solution and substances for the hygiene of dentures had no significance impact in the SFE value. An incubation of retainers in orange juice and coffee influenced increase of the total SFE value (increase of the SFE dispersive component and decrease of polar component) (Table 5). The decrease of the SFE value was observed for retainers incubated in water and saline solution (significant decrease of the SFE polar component).

**Figure 5:**
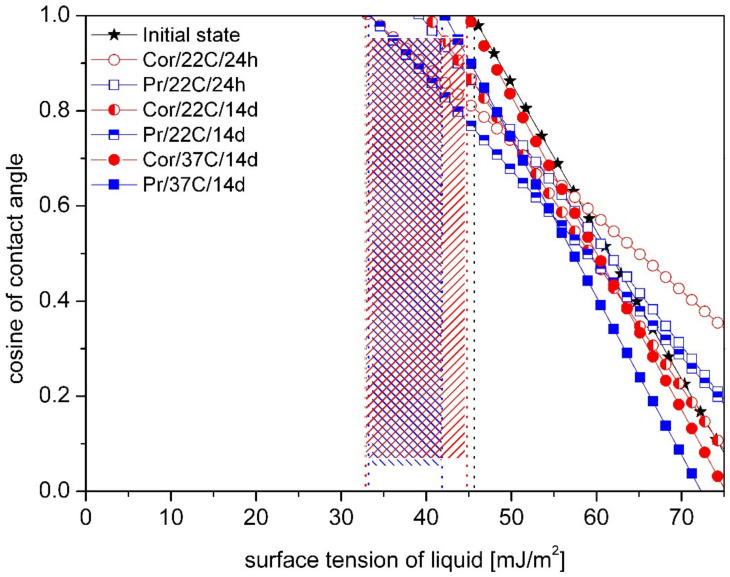
Comparison of the medium influence on the critical surface free energy (marked with vertical lines) for Villacryl H Plus obtained by Zisman’s method. The hatched area shows the difference of γ_C_ between samples in the initial state and after storage.

**Figure 6:**
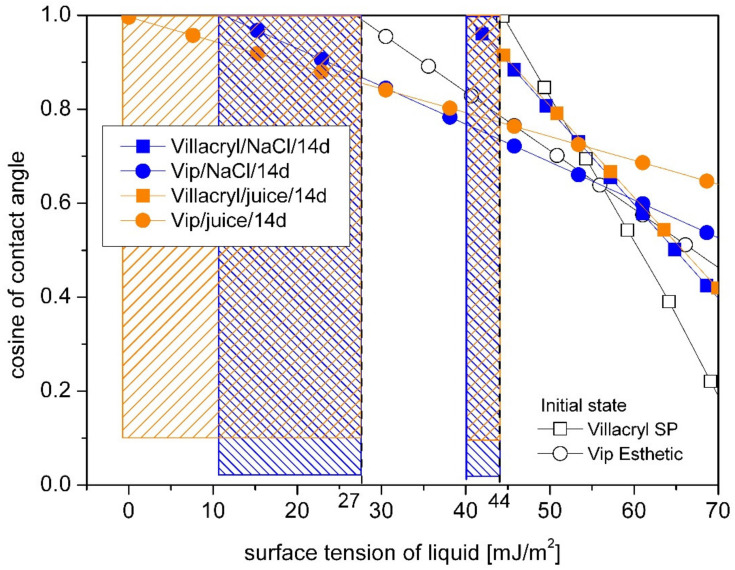
Comparison of time storage and medium type influence on the critical surface free energy (marked with vertical lines) for Vip Esthetic and Villacryl SP dental materials obtained by Zisman’s method. The hatched area shows the difference of γ_C_ between samples in the initial state and after storage.

**Figure 7:**
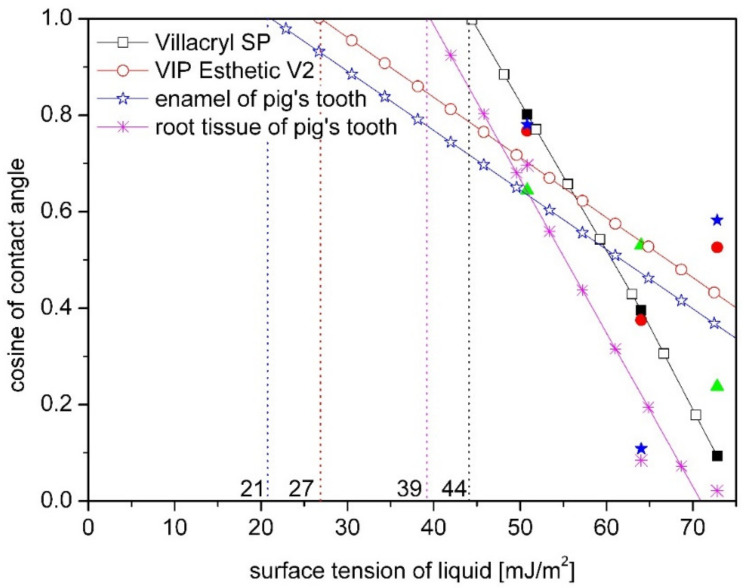
Comparison of the critical surface free energy (marked with vertical lines) for pig tooth, acrylic resin (Villacryl SP) and composite (VIP Esthetic V2) obtained by Zisman’s method.

Zisman’s plots with obtained values of the critical energy for tested materials were shown in figures below, from Figure 5, Figure 6 and Figure 7. Different type of storage medium, time and temperature caused change of the critical energy values for dental material Villacryl H Plus (Figure 5). The initial value of γ_C_ was 46 mJ/m^2^, and the highest decrease was observed after incubation in Prokudent for 14 days in the room temperature (33 mJ/m^2^). Dependence on medium, time and temperature of incubation, decrease from 2.17 to 28.2% of the critical energy value was observed.

Villacryl SP showed similar to Vilacryl H Plus initial value of critical energy (Figure 6). After incubation in saline solution or orange juice, a slight decrease of critical energy value was observed. In comparison to acrylic denture resins, composite (Vip Esthetic) showed significantly lower value of γ_C_ (27 mJ/m^2^). Decrease of this value after incubation was dependent on liquid type with a greater effect of orange juice.

Comparing dental materials with tooth tissues, it was observed that similar like for the SFE value, the value of critical energy is comparable for composite and pig’s enamel (Figure 7).

Analyzing the obtained energy values in terms of bioadhesion determined by Baier [23,28] on the base on Zisman’s plot, it can be noticed that acrylic materials are in the zone of good bioadhesion, while dental tissues and BIS-GMA composite are in the zone of poor adhesion. It influences on materials interactions with biological phases and controlling the surface energies provides possibility to control cell-surface interactions and prevent bacterial adhesion. This issue will be discussed in the next chapter.

## 4. Discussion

Materials with different surface characteristics are used in the oral cavity (teeth, filling materials, dental implants, or prostheses). Conducted tests of the contact angle and calculations of the surface free energy showed the differences in the energy state of dental materials and tissues, as well as the influence of contact with various liquids on the analyzed values. Materials’ surface in biological fluid is deposited by organic films and cells, and in case of dental materials, the primary issue is dental plaque formation. This applies for natural structures as well as for restorative or prosthetic materials. Dental plaque is favored by two mechanisms: adhesion and stagnation. The adherent bacterial biofilm is associated among others with the development of caries, periodontal diseases, peri-implantitis, or denture-associated stomatitis. High-energy surfaces are known to collect more plaque, to bind the plaque more strongly and to select specific bacteria [30]. However, studies examining the relationship between surface wettability and bacterial adhesion show conflicting results. Some studies report that bacterial adhesion is higher on hydrophobic surfaces, and other studies show increased biofilm development for highly hydrophilic substrates [7]. Sang et al. [16] suggested that new dental materials should be designed for controlling bacteria attachment by tuning thickness, composition and structure of the adsorbed salivary pellicle. Cavalcanti et al. [17] compared an uncoated PMMA acrylic resin to PMMA coated with saliva and observed a decreased of the total SFE value from 36.6 ± 2.5 mJ/m^2^ to 31.8 ± 2.5 mJ/m^2^. Additionally, used of the saliva with plasma pellicle impacted significantly on higher total SFE value (43.9 ± 2.2 mJ/m^2^) and greater value of polar component with negative charge (22.5 ± 2.7 mJ/m^2^).

Obtained values of contact angle and surface free energy for light-curing composite are in good agreement with results obtained by Namen et al. [19]. The investigation of SFE and wetting of light-curing polymers by two different system (LED/halogen lamps) showed the values of water contact angle in the range from 59.2 ± 3.9° to 74.4 ± 3.9° and values of SFE from 34.7 ± 2.0 to 40.2 ± 4.8 mJ/m^2^ [19]. Neves et al. evaluated the capacity of sodium hexametaphosphate (HMP) at different concentrations to alter the surface properties of dental enamel in order to increase calcium and phosphate adsorption in tests of wettability. The value of SFE for raw bovine enamel in the initial state was reported at 28.8 ± 4.6 mJ/m^2^, and the water contact angle was 67.7 ± 4.1° [20], which differs from the values obtained for swine tooth enamel.

Conducted studies revealed varying degree of influence of storage in several liquids on contact angle and SFE. Different formulations of the experimental resin-based restorative materials and its influence on wettability (values of water contact angle) and SFE after water storage were tested in [31]. Materials after storage showed increased θ indicating very high hydrophobicity (87.0° ± 3.1° to 110.9° ± 3.5°) in comparison to the dry materials, which had contact angle in range from 56.6° ± 3.3° to 90.5° ± 3.8°. Our studies showed increase of hydrophobicity and decrease of SFE to the value of 28.7–29.4 mJ/m^2^ after storage in water and saline solution for retainers (PET-G). Increase of SFE value after storage in orange juice and coffee for tested retainers indicates increased interactions with these media and is a disadvantageous phenomenon due to possible adhesion of bacteria [31]. Decrease of SFE energy was also observed for denture acrylic resins after storage in substances for the hygiene of dentures, but calculated values of SFE (42.2–46.0 mJ/m^2^) showed hydrophobic character of the surface and may increase bacterial adhesion [31].

Many attempts have been made to modify the chemistry of the dental material and develop procedures to diminish or even inhibit pellicle and bacterial adhesion. With the aim of diminishing plaque formation, different additives were used, e.g., fluoride releasing materials, silver nanoparticles, fluorine polymers and anti-microbial monomers [19,20,31]. Chewing causes abrasion and microleaks of filling and prosthesis; therefore, there are only limited possibilities of keeping the ideally smooth surface of materials in the oral cavity. For this reason, the importance of SFE and wettability for dental materials in the oral environment is increasing. The influence of chewing simulation on wettability and SFE was evaluated in [21]. All tested resin-based dental restorative materials showed decrease of contact angle after chewing simulation. The value of water contact angle for basic material decreased from 57.6 ± 4.3° to 36.9 ± 5.4° and in a group of modified materials decreased from the range of 95.9 ± 4.0° to 105.6 ± 1.2° before chewing simulation and from 59.0 ± 8.4° to 76.3 ± 12.7° after chewing simulation [21]. This reported initial value of contact angle is in very good agreement with the value obtained for composite resin (Vip Esthetic), and influence of storage in medium caused a decline effect of its value but to a lesser extent than chewing simulation. Considering the SFE, authors [21] reported its values at about 44 mJ/m^2^ for basic material, and strong influence of chewing simulation on γ_S_—(increase from 16.2 ± 2.8 to 45.9 ± 7.2 mJ/m^2^). For the group of modified materials, Rutemman et al. obtained the SFE value of about 30 mJ/m^2^ before chewing simulation and in the range from 19.5 ± 12.6 to 33.1 ± 3.1 mJ/m^2^ after simulation [21].

Among tested materials, favorable surface properties with low surface energy were observed only for PET-G used for retainers for which values were in the range of poor bioadhesion zone. Other tested materials were characterized by the values of critical surface energy above 40 mJ/m^2^, which indicates the range of good bioadhesion and may provide attachment of fouling debris [28,29]. 

The understanding of the complex interactions between oral microorganisms and resin-based composite materials could be of great importance to guide the development of new materials able to modulate microbial adhesion and biofilm formation [12,18].

## 5. Conclusions

Surface characteristic is a fundamental step in testing materials to be used in the biological field. This study focused on estimation of adhesive effects based on the surface free energy, critical surface tension and wetting properties of dental materials. Importantly, these properties were assessed on the basis of comprehensive analysis. Measurements of contact angle were conducted with the use of several liquids, not just water. Biological adhesion is associated with defined ranges of surface free energy or critical surface tension. Obtained results showed that materials used as restorative, denture or orthodontic dental materials have different adhesion capacity. A contact angle and SFE can be used to show differences in surface properties of dental materials. Comparing energetic state of different dental materials and identifying those with high value of surface free energy can be a practical guide to avoiding such materials when the conditions for maintaining oral cavity hygiene are unfavorable. Different dental materials treated with varying storage conditions gave basis to compare surface energy and wettability between the initial materials’ surface and changed by the action of storage conditions. This is an additional value of the analysis due to the fact that water is most often used as an environment in other studies. The wetting of a surface by a liquid and the ultimate extent of spreading of that liquid are very important aspects of practical surface chemistry, and there is still a great deal to learn about the mechanisms of movement of a liquid across a surface.

## Figures and Tables

**Table 1:** Dental materials used in tests.

Material	Basic Composition, Application	Manufactures
Villacryl SP	cold-curing acrylic resin for frameworks, making partial and full prostheses by pouring in hydrocolloids, mask silicones and duplicating silicones; it can also be used for repairs and for indirect relining	Zhermack, Italy
Villacryl H Plus	heat-curing acrylic resins, specially formulated for denture bases, removable full or partial prostheses and for the indirect relining of removable prostheses	Zhermack, Italy
Villacryl S V4	self-curing acrylic resin for the repair and indirect relining of removable prostheses (color pink veined)	Zhermack, Italy
VertexTH Self-Curing	self -polymerizing cold-curing acrylic material used both for repair and relining of full and partial dentures	Vertex-Dental, The Netherlands
Flexite T-Val	polyolefin thermoplastic for flexible partial dentures	Flexite Company, USA
Vip Esthetic	BIS-GMA- light curing resin and inorganic filler particles od 0.05–1.5 μm, suitable for all cavities	Olident, Poland
Dental retainer	Duram^®^+—polyethylene terephthalate glycol-modified (PET-G) used for dental retainers	SCHEU_DENTAL GmHb, Germany

**Table 2:** Conditions of samples incubation.

Material	Environment	Temperature [°C]	Time
Villacryl SP	0.9% NaCl / Orange juice (pH 3.5)	37	14 days
Villacryl H Plus	Corega (fixing cream 1.3 g + 40 mL of 0.9% NaCl)	22/37	24 h/14 days
	2. Prokudent (cleaning substance 3.318 g + 40 mL of water)	22/37	24 h/14 days
Vertex TH Self-Curing	0.9% NaCl	37	21 days
Flexite T-Val	0.9% NaCl	37	21 days
Vip Esthetic	0.9% NaCl / Orange juice (pH 3.5)	37	14 days
Dental retainer	water (pH 4.9)/orange juice (pH 3.5)/coffee (4.8 g + 100 mL water) pH 4.9)/0.9% NaCl	37	60 days
Swine molar	0.9% NaCl	37	24 h

**Table 3:** Values of the water contact angle for tested materials.

Material	Contact Angle (°)	
	Initial State	After Incubation
Swine tooth enamel	50.2 ± 17.6	54.4 ± 17.0
Swine root tissue	62.1 ± 7.7	88.8 ± 9.3
Villacryl SP	84.72 ± 3.79	65.48 ± 3.43 in 0.9 NaCl
		65.01 ± 3.37 in orange juice
Vip Esthetic	58.8 ± 4.1	58.4 ± 3.6 in 0.9 NaCl
		49.1 ± 5.7 in orange juice
Villacryl H Plus	77.6 ± 6.0	85.3 ± 5.0 in Corega, 24 h
		69.1 ± 4.5 in Prokudent, 24 h
		81.3 ± 6.9 in Corega, 14 days
		60.9 ± 4.7 in Prokudent, 14 days
Vertex Self Curing	76.4 ± 2.8	71.2 ± 0.6
Flexite T-Val	75.0 ± 3.7	69.9 ± 2.2
Dental retainer	81.9 ± 1.8	99.6 ± 4.5 in 0.9% NaCl
		95.6 ± 4.3 in water
		83.6 ± 6.3 in orange juice
		85.4 ± 2.9 in coffee

**Table 4:** Comparison of the SFE and its components (mJ/m^2^).

Material	Owens–Wendt			Van Oss–Chaudhury–Good				
	$\gamma_{S}$	$\gamma_{S}^{d}$	$\gamma_{S}^{p}$	$\gamma_{S}$	$\gamma_{S}^{LW}$	$\gamma_{S}^{AB}$	$\gamma_{S}^{+}$	$\gamma_{S}^{-}$
Pig tooth/ enamel *	55.57 ± 8.70	40.26 ± 3.94	15.31 ± 8.92	74.88 ± 6.79	40.26 ± 3.94	34.62 ± 3.75	4.97 ± 4.96	60.27 ± 5.87
Pig tooth/ root *	38.06 ± 5.55	36.50 ± 4.88	1.56 ± 1.45	40.11 ± 8.49	36.50 ± 4.88	3.61 ± 0.61	0.53 ± 0.02	6.22 ± 0.22
Villacryl SP	43.09 ± 3.79	41.22 ± 3.15	1.87 ± 0.52	42.83 ± 10.21	41.22 ± 13.15	1.61 ± 0.77	0.37 ± 0.11	1.76 ± 0.85
Villacryl SP *	49.90 ± 1.07	42.58 ± 1.12	7.32 ± 0.05	44.02 ± 9.88	42.58 ± 11.12	1.44 ± 0.82	0.04 ± 0.01	16.02 ± 3.66
Villacryl SP **	49.96 ± 0.77	40.10 ± 1.22	9.87 ± 0.05	40.62 ± 11.72	40.10 ± 10.22	0.53 ± 0.02	0.01 ± 0.01	19.52 ± 4.95
Vip Esthetic	52.79 ± 3.82	39.66 ± 2.39	13.13 ± 3.18	45.43 ± 16.92	39.66 ± 2.39	5.77 ± 0.53	0.26 ± 0.09	31.77 ± 7.46
Vip Esthetic *	51.15 ± 7.48	36.47 ± 0.15	14.68 ± 1.50	41.80 ± 8.39	36.47 ± 9.25	5.33 ± 2.54	0.45 ± 0.12	23.29 ± 3.63
Vip Esthetic **	54.65 ± 8.15	36.74 ± 0.26	17.91 ± 4.10	42.92 ± 10.02	36.74 ± 9.26	6.18 ± 1.83	0.33 ± 0.03	29.31 ± 4.23
Villacryl S V4	43.55 ± 2.07	42.08 ± 1.32	1.47 ± 0.75	-	-	-	-	-
Villacryl H Plus	48.07 ± 3.02	44.77 ± 1.18	3.31 ± 0.62	47.05 ± 3.91	44.77 ± 1.18	2.28 ± 0.11	0.15 ± 0.05	8.75 ± 0.63
Villacryl H Plus (Corega 22°C/24 h)	42.20 ± 1.55	40.91 ± 0.83	1.92 ± 0.88	46.04 ± 3.42	40.91 ± 0.83	5.77 ± 0.11	0.97 ± 0.07	8.65 ± 0.01
Villacryl H Plus (Corega37C/14d)	45.98 ± 3.43	43.58 ± 1.82	2.40 ± 1.73	46.44 ± 4.63	43.58 ± 2.06	2.85 ± 0.78	0.28 ± 0.08	7.38 ± 0.47
Villacryl H Plus (Prokudent22°C/24 h)	49.14 ± 2.90	41.71 ± 1.45	7.43 ± 1.89	53.21 ± 10.38	41.71 ± 1.45	11.51 ± 0.37	2.03 ± 0.60	24.74 ± 9.15
Villacryl H Plus (Prokudent37°C/14 d)	53.10 ± 3.42	41.95 ± 1.27	11.15 ± 2.82	50.70 ± 10.09	41.95 ± 1.27	8.85 ± 0.58	0.64 ± 0.04	30.51 ± 1.51
Vertex Self Curing	44.79 ± 1.52	40.11 ± 0.79	4.69 ± 0.97	-	-	-	-	-
Vertex Self Curing *	43.81 ± 0.64	36.81 ± 1.61	8.00 ± 0.32	-	-	-	-	-
Flexite T-Val	46.56 ± 2.53	40.84 ± 1.00	5.72 ± 2.36	-	-	-	-	-
Flexite T-Val *	41.76 ± 1.30	31.7 ± 1.61	10.09 ± 1.49	-	-	-	-	-

* sample incubated in 0.9% NaCl, ** sample incubated in orange juice.

**Table 5:** The influence of medium type on the values of SFE and its components according to the Owens–Wendt model for dental retainers.

Medium	$\gamma_{S}$	$\gamma_{S}^{d}$	$\gamma_{S}^{p}$	$\gamma_{S}$	$\gamma_{S}^{d}$	$\gamma_{S}^{p}$
	(mJ/m^2^)					
	Initial State			After Incubation		
dry/ orange juice	33.7 ± 2.7	28.1 ± 3.4	5.6 ± 1.1	38.9 ± 2.3	35.9 ± 1.9	3.1 ± 0.9
dry/ coffee	32.21	25.05	7.17	41.1 ± 1.8	39.0 ± 1.3	2.0 ± 0.6
dry/0.9% NaCl	32.67	27.88	4.76	29.4 ± 1.7	28.8 ± 1.6	0.5 ± 0.1
dry/ water	32.25	26.68	5.57	28.7 ± 0.9	27.2 ± 1.3	1.4 ± 0.2

## Data Availability

Data sharing is not applicable.
